# Crop production in Russia 2030: Scenarios based on data from the scientific and technological development of the sector

**DOI:** 10.1016/j.dib.2019.103980

**Published:** 2019-05-09

**Authors:** Evgeni Vladimirovich Rudoi, Marina Sergeevna Petukhova, Sergey Vladimirovich Ryumkin, Svetlana Leonidovna Dobryanskaya, Alexandra Valeryevna Molyavko

**Affiliations:** Branch Center for Forecasting and Monitoring of Scientific and Technological Development of Agroindustrial Complex, Novosibirsk State Agrarian University, Novosibirsk, Russian Federation

## Abstract

A feature of modern crop production is the acute need to accelerate its scientific and technological development, on the basis of innovative processes. The sector of crop production has an essential dependence on external factors and the modern directions in its scientific and technological development should also reduce dependence on external factors and to improve controllability by reducing the uncertainty of responses to external influences. The methodology of scenario forecasting, adapted to the crop production gives the opportunity to answer the questions such as, for example as:

How the determinants of the development of the crop sector will change? What future bifurcation points may occur? What strategic decisions can be made? What consequences these decisions will bring in future?

Among the stages of long-term forecasting, the special part is assigned to development of scenarios of development. Scenario prediction allows, based on the available data, to suppose the development and behavior of the object under study in the future. As a result, it becomes possible to develop strategic and tactical solutions based on the implementation of the proposed scenarios. The peculiarity of this method is that it is applicable in situations of uncertainty of the object's reactions to various external influences.

The development of scenarios allows to surmount the stochastic nature of the processes occurring in the scientific and technological sphere, to expose large-scale scientific and technological breakthroughs that can significantly change the crop sector. Scenario approach as much as possible forces out uncertainty of choice space between scenarios.

**Table:** Specifications table

Subject area	*Economy, Agriculture, Crop production*
More specific subject area	*Agronomy, Organic farming, Seed growing*
Type of data	*Text, table, figures and graphs*
How data was acquired	*The data of the bibliometric analysis are based on the Web of Science database (ResearcherID), search by keywords related to agrobiotechnology. Informative data are obtained from Forecasts of scientific and technological development of Russia: 2030*
Data format	*Processed and analyzed data and information*
Experimental factors	*Formation of scenario development based on data of world trends in plant growing, seed farming and organic farming, studied using bibliometric analysis*
Experimental features	*Through the scenario forecasting of the scientific and technological development of the crop industry, the possibility of a vision of the future is identified, and an algorithm for developing scenarios “Technological adaptation” and “Technological breakthrough” is defined*
Data source location	*Data on the crop sector of the Russian Federation*
Data accessibility	*The data set is presented in the proposed article and is available by reference*
Related research article	*This article is a continuation of the research “Methodical approaches to forecasting the scientific and technological development of the crop sector” by E.V. Rudoy, S.V. Ryumkin, M.S. Petukhova and others (Achievements of science and technology of agroindustrial complex 31(10) (2017) 8–17)* *“Forecasting the development of markets for critical technologies in the crop sector until* 2030/E.V*. Rudoy, M.S. Petuhova, R.R. Galeev* et al.*//Achievements of science and technology of agro-industrial complex. - 2018. - Vol. 32. - No. 4. - P. 5–9. “*

**Table:** 

**Value of the data** • The set of bibliometric data presented in the article allows to examine in more detail the directions of global trends, as well as to study the views and technologies of scientists working in the crop sector. • The algorithm for the development of the scenario conditions makes it possible to determine the goals, objectives and priorities of the medium and long-term prospects for the development of the crop sector. • The presentation of two scenarios of the scientific and technological development of the crop sector “Technological adaptation” and “Technological breakthrough” gives the opportunity to define the level of scientific and technological development of the internal and the world markets. • The system of indicators and target indicators characterizes the scientific and technological development, its further dynamics and contributes to the formation of a scenario for further development in the short term.

## 1 Data

The formation of scenarios occurs under the influence of global trends [1], [2], [3], [4], [5], [6], [7], [8], [9], [10], [11], which are responses to global challenges (Table 1).

**Table 1:** Global trends affecting the development of the crop sector.

Type	Challenges
Economic	• the growth in global demand for organic and organic crop products; • the growth rates of population is ahead growth rates of world gross production of crop production;
Technological	• reduction of crop areas of grain crops in the USA, Canada, China; • exhaustion of the potential of the “green revolution"; • reduction of the natural breed and variety biodiversity in crop production.
Environmental	• global warming; • reduction of natural soil fertility, erosion; • mass deforestation;
Social	• differentiation of incomes of the population; • an increase in the pace of urbanization.

The given data testifies to the global, annually accumulating problems that directly affect the development of the crop industry in Russia. So economic trends lead to the fact that the crop industry needs to be developed along the organic path, excluding the use of mineral fertilizers and toxic chemicals. In this case, government support for the crop industry will be directed to ensuring the production of organic products. Among technological trends, the greatest impact on the development of the crop industry in Russia is exerted by a worldwide reduction in acreage and a decrease in high-quality biodiversity. Along with the global environmental problems, this will allow the crop industry in Russia to maximize the use of available acreage with the use of world technological innovations in the field of urbanized crop production.

**Fig. 1:**
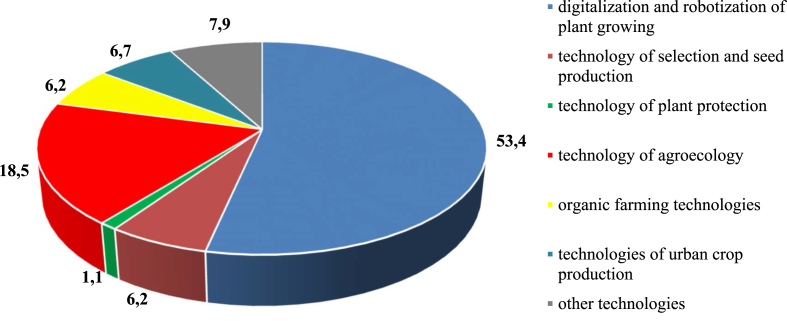
The diagram of the distribution of keywords in the database Web of Science (ResearcherID), associated with agrobiotechnology in crop production, %. Of the 178 analyzed Russian and foreign publications on crop production technologies, the following areas are of the greatest interest: 53.4% precision farming, remote sensing, 3D crop modeling, robotization; 18.5% - agroecology and agroforestry system; 7.9% - organic waste, peat compost; 6.7% - hydroponics, aeroponics, vertical greenhouses; 6.2% - cell selection, genomic selection, agricultural genomics; 6.2% - soil remediation, screening of microorganisms, adaptive landscape system of agriculture; 1.1% - plant protection products, nutrient solution, integrate pest management.

The global trends were conducted based on bibliometric analysis, which was carried out in the database of Web of Science (ResearcherID), by keywords related to agrobiotechnology (Fig. 1).

## 2 Experimental design, materials, and methods

### 2.1 Methodology of scenario forecasting in the field of crop production

Scenario forecasting is a tool that works in conditions of high uncertainty of the future in the medium and long term, allowing to identify the most likely variants of the development of events [9], [10].

Scenario forecasting of scientific and technological development of crop production is intended for:

• creating a common vision of the future of all key players in the crop sector and determining the place the crop production in the economy of the country wants to occupy in the future;
• understanding and visualizing the consequences of decisions made today, for scientific and technological development of crop production;
• visualization of all possible options for scientific and technological development of crop production.

**Fig. 2:**
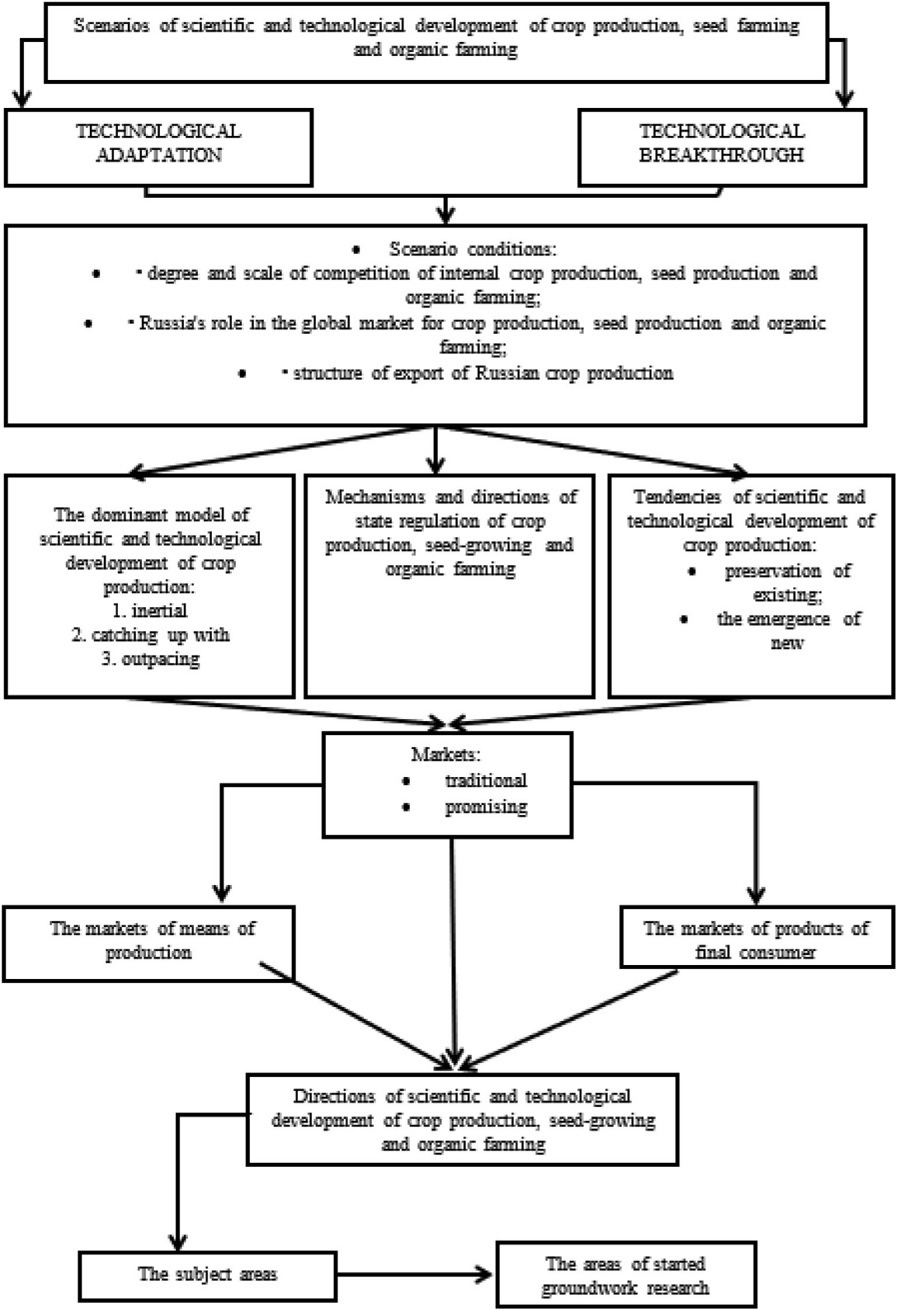
Algorithm for the development of scenarios for the forecast of scientific and technological development of crop production, including seed farming and organic farming (developed by the authors).

Scenario forecasting of the scientific and technological development of crop production requires the definition of a step-by-step algorithm for constructing the forecast, starting with the identification of possible scenarios and ending with the areas of started groundwork research for each of the scenarios (Fig. 2).

The two proposed scenarios should be aimed at achieving the main goal of scientific and technological development of domestic crop production - ensuring the competitiveness of Russian products in the domestic and foreign markets, primarily through the creation and introduction of the latest achievements in science and technology [11].

The algorithm for developing scenarios for forecasting the scientific and technological development of crop production, including seed production and organic farming, contains the following stages:

1. Identification of two main scenarios:
  • “Technological adaptation” - preservation of existing trends in scientific and technological development, stable positive dynamics of production of the majority of agricultural crops, import of basic technologies of crop production, seed-growing and organic farming, expanded reproduction of crop production through predominantly extensive factors;
  • “Technological breakthrough” - achievement of leadership positions in certain areas of scientific and technological development of crop production, seed farming and organic farming, and changes in the structure of cultivated and exported crops, expansion of crop production on an innovative basis.

**Fig. 3:**
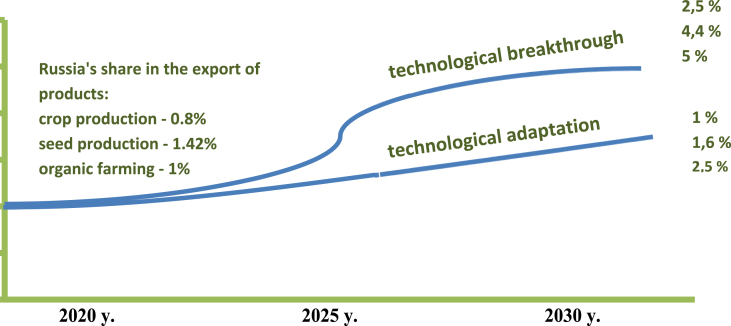
The process of implementing the scenarios “Technological adaptation” and “Technological breakthrough". If current trends continue, the share of Russian exports in the global market by 2020 will be: crop production - 0.8%; for seed production - 1.42%; organic farming products - 1%. To strengthen its position in the global agrifood market of Russia, it is necessary to use the opening windows of opportunities, in this case, by 2025, when implementing the “Technological adaptation” scenario, Russia's share will be: crop production - 1.0%; for seed production - 1.6%; organic farming products - 2.5%. While maintaining the pace of development, the “Technological adaptation” scenario will set in motion the mechanisms for launching the “Technological breakthrough” scenario and by 2030 Russia's share on the global agrifood market will be: crop production - 2.5%; for seed production - 4.4%; organic farming products - 5%.2.Definition of scenario conditions is the basic prerequisites for the scientific and technological development of crop production, seed production and organic farming, including priorities, goals and objectives for the medium and long term [12], [13]. These include the degree and scale of the competition of internal crop production, the role of Russia in the world market of crop production; structure of export of Russian crop production, etc.3.For each scenario it is necessary to allocate:•the dominant model of scientific and technological development of crop production, seed farming and organic farming: inertial (maintaining the existing dynamics of scientific and technological development of crop production), catching up with (the main goal is to overcome the backlog of the crop sector according to the level of technological development of the leading countries) or the outpacing (accelerated technological growth of crop production in the medium and long term, due to qualitative changes in the institutions and structure of the economy, in order to outpace the scientific and technological development of the crop production among the leading countries);•mechanisms and directions of state regulation of crop production, seed farming and organic farming, for example, financing of strategically important scientific and technological projects, training highly qualified specialists, stimulating import substitution, etc.•trends in the scientific and technological development of crop production: will the current trends be preserved (a moderate increase in the technical and technological equipment and productivity of agricultural producers) or will be new (robotization, computerization, export orientation)?4.The implementation of scenario conditions will lead either to the expansion of traditional markets or to the emergence of the new ones. In particular, the scenario “Technological adaptation” presupposes strengthening of Russia in the traditional market of crop production and means of production, and the scenario “Technological breakthrough” - the emergence of markets for organic and urban crop production, technologies for precision farming and other digital technologies.

At the same time, it should be noted that in the “pure form” these scenarios will not be realized and the scenario “Technological adaptation” will become the basis and the initial stage of the scenario “Technological breakthrough”. The latter will be implemented gradually, only after the first scenario is implemented completely (Fig. 3).

For each scenario, their markets will be allocated: means of production, platform solutions, final consumption products.

5. The result of scenario forecasting will be the formulation of areas of scientific and technological development of crop production, seed production and organic farming, that is, a list of critical technologies that will significantly increase the efficiency and productivity of the crop sector. In this case, it is necessary to take into account the market component of this process: the created technologies can appear not demanded in the world market. Therefore, scenarios are developed on the principle “from markets to technologies”, because it allows to identify the most critical technologies in the future. After determining the prospective market niches for internal crop production, it is necessary to identify areas of counterfeit research, where the country's resources should be concentrated to obtain technologies that have long-term competitive advantages.

#### 2.2 Scenario conditions for forecasting the scientific and technological development of the crop sector

Close attention should be given to scenario conditions since they set the initial vector for future scenarios of scientific and technological development.

The approach to the definition of scenario conditions is set by the requirements of the Resolution of the Government of the Russian Federation, July 13, 2015 No. 699 “On Approving the Rules for Development and Correction of the Forecast of the Scientific and Technological Development of the Russian Federation” and Order No. 1335 of the Ministry of Education and Science of the Russian Federation, November 13, 2015 ″ On the approval of methodological recommendations on the preparation of initial data for the development and correction of the forecast of scientific and technological development of the Russian Federation, as well as on the formation of its scenario conditions " [12].

Scenario conditions should take into account the priorities, goals and objectives of the sector's development: the primary goal of the scientific and technological development of the agro-industrial complex of the Russian Federation in the future is to ensure the competitiveness of Russian products in the external and internal markets, primarily through the creation, dissemination and application of the latest achievements in science and technology [12], [14].

The scenario conditions and the main parameters of the forecast contain a description of the conditions, characteristics and indicators of the scientific and technological development of the Russian Federation for a long-term period and are developed for a period of up to 30 years (with the selection of periods) in conjunction with the parameters of the long-term socio-economic development forecast of the Russian Federation [12].

The structure of the scenario conditions and the main parameters of the forecast are as follows:

a) external conditions, including the main global trends of scientific and technological development;
b) internal conditions, including trends in scientific and technological development in the Russian Federation;
c) the achieved level of scientific and technological development of the Russian Federation, including in comparison with world trends;
d) factors of scientific and technological development of the Russian Federation (macroeconomic, structural, institutional, etc.);
e) challenges and threats to the Russian Federation (economic, environmental, technogenic, demographic, etc.).

The identification of scenario conditions and basic parameters allows to form:

• Scenarios of scientific and technological development, their description and main characteristics, corresponding to the goals and opportunities of scientific and technological development of the Russian Federation in the forecast period [10], [15];
• Key indicators of the forecast of scientific and technological development of the Russian Federation, characterizing the state of scientific and technological development for a long-term period.

Together with scenario conditions, the criteria for selection of promising markets are formed, including groups of innovative products and services, technologies, research areas for developing forecasts for the technological development of economic sectors and forecasting the scientific and technological development of the Russian Federation [16].

The conditions for the implementation of the scenario “Technological adaptation” is to going to consist in stable positive dynamics of production of most agricultural crops, in satisfying the Russian market with seeds of domestic production, etc. Economic growth will be achieved through the expanded reproduction of agricultural producers (development of extensive factors in combination with modernization). This will allow them to master the internal market and increase the export of crop production in some areas. Basically, traditional markets for technologies and crop production will be developed. Agricultural producers will not change their preferences and mainly imported machinery and equipment will be used in production.

As for selection and seed production, the continuation of the policy of import substitution will create conditions for the satisfaction of the internal market with internal seed.

Organic farming will develop on a limited scale, as will the demand for it within the country, so the sale of products will be mainly carried out to foreign markets. At the present time, there is a problem of inadequate information among agricultural producers on the principles of organic farming, which, within the framework of this scenario, will be gradually addressed through raising their awareness and interest in the production of organic crop production. There will also be an increase in farms producing organic products that meet international standards.

The main purpose of the scenario “Technological breakthrough” is to try to outstrip the crop sector with the newest technological base, corresponding to the decisions of the “tomorrow's” day. The conditions of this scenario are a change in the structure of the cultivated crops and their exports, complete import substitution of seed material, etc. There will be a significant diversification of marketable products in the direction of reducing the share of grain crops. The expansion of production under this scenario will take place on an innovative basis, through the mass introduction of new crop production technologies into production. Due to the outstripping rates of economic growth, the scientific and technological backlog of the crop sector will be diminished in comparison with the advanced foreign countries, moreover, dependence on imported technologies will be also decrease.

#### 2.3 The system of indicators of the scenario forecast of the scientific and technological development of the crop production

The implementation of any scenario of scientific and technological development of the crop sector is carried out through the achievement of various indicators associated with the crop sector and characterizing the scenario. Each of the scenarios is characterized by a different level of achievement of goals, which can be absolute and relative. The gap between the indicators allows to determine the degree of impossibility achieve one scenario of another.

The system of indicators allows to determine the current state of the scientific and technological development of the crop sector, and an analysis of their dynamics makes it possible to determine the direction of development. It is necessary to identify such indicators that will help to identify and understand which of the proposed scenarios is most likely in the near future.

As for Russia, the authors of the article refer to such indicators:

1. The average annual growth rate of the gross output of the following crops: wheat, rye, barley, oats, corn, rice, rapeseed, sunflower, soybeans, sugar beet, potatoes, and protected ground and open ground vegetables.
2. Specific weight:
  • grain crops in the structure of production and export of crop production;
  • Russia in the structure of world exports of crop production.
  • use of internal seeds in the production of crop production;
  • products of organic farming in general crop production;
  • organic farming products that meet international standards;
  • internal crop production technologies in the internal market, including the technologies of precision farming, urban agriculture, organic farming, etc.
3. A number of certified organic producers in the Russian Federation.
4. Area of certified land for organic farming.
